# Polyphenols and exercise in autophagy regulation: potential benefits for cancer management and healthspan

**DOI:** 10.3389/fnut.2025.1618813

**Published:** 2025-09-08

**Authors:** Wei Liu, MengDi Hu, Feng Cao, Shen Jin

**Affiliations:** ^1^Department of Physical Education, Xidian University, Xi’an, Shaanxi, China; ^2^Shaanxi Provincial Hospital of Chinese Medicine, Xi’an, Shaanxi, China; ^3^Ersha Sports Training Center of Guangdong Province, Guangzhou, Guangdong, China

**Keywords:** polyphenols, exercise, autophagy, cancer, oxidative stress, metabolic health

## Abstract

Autophagy, a regulated cellular process, serves as both a tumor suppressor and a survival mechanism for tumor cells under stress in cancer. Recent studies demonstrate that polyphenols, bioactive compounds present in plant-derived foods, and exercise, a potent physiological stimulus, can efficiently modulate autophagy in both cancer patients and healthy individuals. This review explores the synergistic effects of polyphenols and exercise in regulating autophagy through key molecular pathways, including AMPK/mTOR, PI3K/Akt, and SIRT1/FOXO. Polyphenols such as quercetin, resveratrol, and curcumin possess autophagy-inducing properties, which may enhance exercise-induced cellular adaptations, contribute to cancer prevention, and improve metabolic health. Moreover, regular physical activity promotes autophagic flux, reducing oxidative stress, inflammation, and apoptosis resistance—factors critical in cancer progression and overall health maintenance. The review highlights the potential of polyphenol-exercise synergy in modulating autophagy, which may result in innovative therapeutic approaches for cancer treatment and metabolic health.

## 1 Introduction

Organisms necessitate fitness, resilience, and adaptability for optimal health, while life sciences and medicine strive to enhance health and avert disease through systematic physical exercise (1). Exercise is essential in rehabilitation medicine for complete recovery from illnesses; however, the molecular mechanisms that facilitate health improvements are poorly understood, especially concerning its effects on cellular processes in different organ systems (2–4). Autophagy is a vital cellular process that breaks down and recycles intracellular components to maintain homeostasis (5–7). It encourages general health, disease prevention, differentiation, development, and survival. Given its critical role in cellular health, dysregulated autophagy is associated with several pathological conditions, including cancer, that indicate possible therapeutic applications by direct targeting of autophagy-related proteins (8, 9). Autophagy, an essential cellular protective mechanism, is being investigated as a potential treatment for cancer via pharmacological agents or dietary modifications.

Autophagy is pivotal in cancer, facilitating both the induction and suppression of tumor growth. It can inhibit inflammation, avert mutations, and forestall chronic tissue damage. Autophagy is crucial for the survival of tumor cells under conditions of cellular stress. Tumor cells lacking autophagy exhibit a survival disadvantage under metabolic stress. Oncogenic pathways enhance autophagy, elevating cellular energy expenditure and facilitating survival. Although autophagy is a non-pharmacological intervention induced by physical exercise, it is still unknown what molecular mechanisms govern autophagic flux and how they might be used to treat cancer (5, 10–12). Drugs that modulate autophagy, such as rapamycin, carbamazepine, cisplatin, and chloroquine, have received approval for human clinical application. Nonetheless, their specificity and organ/cellular selectivity constrain their applicability. Natural products, including polyphenols present in fruits, herbs, vegetables, tea, wine, and cereals, present a promising therapeutic approach for regulating autophagy. Despite their well-established antioxidative properties, current research has indicated other mechanisms through which polyphenols exhibit health benefits. The regulation of autophagy by polyphenols to benefit human health by exercise has become an important new area of research focus. The relationship between exercise and polyphenols in regulating autophagy for cancer recovery is still ambiguous, despite extensive research on their advantages (13, 14). This review analyzes the potential advantages of exercise and polyphenol-induced autophagy in enhancing well-being and preventing cancer. It investigates the molecular mechanisms, effects on particular tissues, and prospective future therapies utilizing polyphenols as chemoadjuvants.

## 2 Protein deficiencies in autophagy: mechanisms, diagnostics, and pathological implications

This section examines autophagy, encompassing chaperone-mediated, microautophagy, and macroautophagy, along with their mechanisms, diagnostic methods, and the effects of protein deletion on cellular homeostasis and human health (15). It underscores the significance of autophagic flux in preserving cellular homeostasis, averting disease, and offering therapeutic advantages, including the synthesis and lipidation of LC3 (16–18). Monitoring the accumulation of microtubule-associated protein 1A/1B, Beclin1, Atg7, and Light Chain 3-II, essential autophagy proteins, is a prevalent technique for assessing flux (19, 20). Deficiencies in proteins such as Atg7 can exacerbate neurodegenerative diseases like Alzheimer’s, promote muscle atrophy, osteoporosis, and cognitive deterioration, underscoring the necessity to comprehend their physiological implications. Deficiencies in autophagy-related 5 can result in heart failure and cardiomyopathy (21, 22). The balance of bone tissue is upset by optineurin deficiency, which results in osteoporosis. Disruption of autophagy-related genes can lead to neurodegeneration, musculoskeletal disorders, cardiovascular problems, and metabolic dysregulation (Table 1). To prevent and treat diseases, it is essential to have optimal autophagy function (23).

**Table 1:** The influence of exercise training on the alteration of crucial autophagic and associated proteins.

Autophagic or-related proteins	Molecular mechanism	References
LAMP2a	Induced the alterations specific to the WAT type	(135)
Atg5	Enhanced mitochondrial function, increased muscle mass, and stimulation of mitophagy. The reduced adiposity and longevity enhancement in Atg5 transgenic mice preserve the homeostasis of aging skeletal muscle cells. Mitigating neurological disorders and preserving neuronal well-being Reducing excessive glycolytic metabolism in the brain	(45, 136–140)
Beclin1	Augmented deposition of amyloid-beta plaques Modifications of microglial cells Enhanced the regulation of autophagy and mitophagy capacity Enhance early myocardial protection and mitigate the risk of myocardial ischemic–hypoxic injury due to extended exercise. Alterations in gene expression during cellular reprogramming, activation of Wnt signaling pathways, and diminishment of scar size post-myocardial infarction	(141–144)
PGC-1α	The adaptive genetic responses of autophagy proteins in skeletal muscle Minimal effects on the hepatic autophagy and mitophagy.	(145–147)
Atg7	Improved the overall metabolism, at least in part, through a heart-brown fat interaction mediated by FGF21 in exercise-trained Atg7h&mKO mice Significant mitochondrial membrane depolarization in skeletal muscle Induced alterations specific to WAT type	(135, 148, 149)
p62	Induced alterations specific to WAT type Improves insulin resistance and slows the advancement of NASH mitigates muscle atrophy in LLC-induced cancer cachexia primarily through Nrf2 activation.	(135, 150, 151)
LC3-II	The alterations to the WAT type entailed transforming glycolytic type IIX muscle fibers into oxidative type IIA fibers, thereby alleviating exercise-induced myocardial ischemic/hypoxic injury.	(135, 152, 153)
TFEB	The nuclear translocation of TFEB in skeletal muscle is initiated by calcineurin-mediated dephosphorylation.	(154)
FOXO	The regulation of this component, potentially affected by Akt/AMPK, may influence the transcriptional regulation of autophagy components	(155)
BCL2	Regulation of stimulus-induced autophagy and glucose metabolism	(156)
mTOR	Potentially facilitating additional stimulation of protein synthesis at subsequent time points	(157)
AMPK	Autophagy activation transpires in reaction to diminished intracellular energy charge, serving as an intracellular energy sensor.	(136)
BNIP3/NIX	The direct engagement with LC3-II is essential for mitophagy, the selective autophagic removal of impaired mitochondria.	(158)
ULK1	Improved mitochondrial selective autophagy and cellular viability during periods of starvation.	(159)

## 3 Exercise-induced autophagy’s signal transduction mechanisms

**Figure 1:**
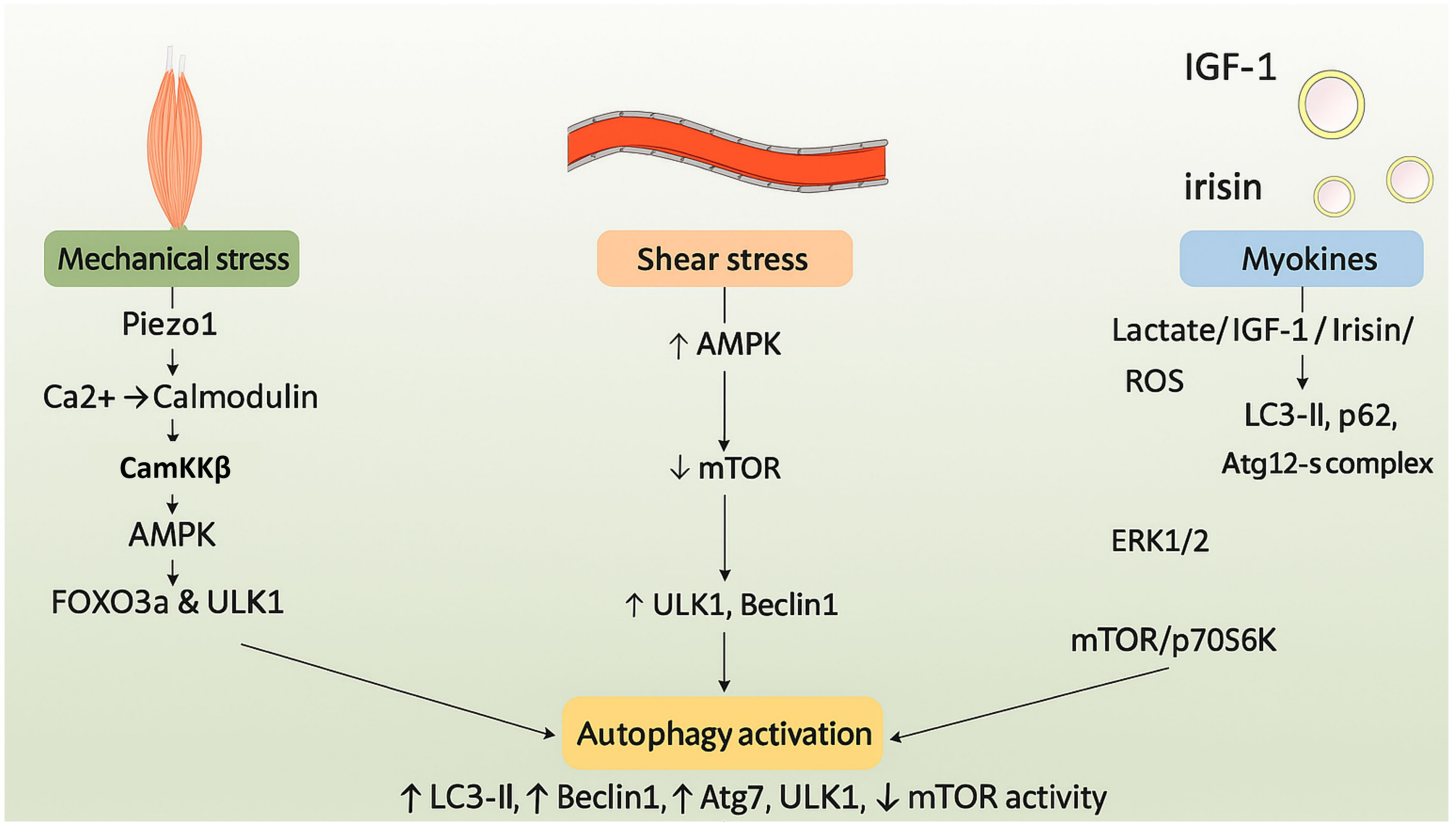
Schematic illustration of the molecular pathways through which exercise induces autophagy. Mechanotransduction and myokine signaling activate AMPK and downstream effectors such as CaMKKβ and FOXO3a. These events suppress mTORC1 and initiate autophagy through ULK1, Beclin1, and LC3 pathways.

Exercise affects the body in two ways: through mechanical forces and biochemical pathways. This has an impact on autophagy in several systems. Mechanotransduction is an important part of the process. It involves the contraction of skeletal muscles, the compression of joints, and the shear stress of blood flow (24). Myokines, which have anti-inflammatory qualities, control metabolism, and affect general health, are also produced in greater quantities when one is physically active. The mechanisms linking exercise to autophagy are examined in this section (25) (Figure 1).

### 3.1 Mechanochemical transduction

Mechanochemical transduction is the process by which external mechanical stimuli are transformed into bioelectric signals within cells. This mechanism influences exercise-induced autophagy through intracellular signaling pathways, cell membrane receptors, and ion channels (26, 27).

### 3.2 Poezol and AMPK: in-depth mechanical forces transmission

Physical activity stimulates mechanosensitive proteins in cells, converting mechanical signals into biochemical responses and altering cell membrane properties, facilitating touch, pain perception, and proprioception (28). Excessive mechanical stress elevates Piezo1 expression, impeding autophagy and hastening intervertebral disc degeneration, whereas AMPK activates calcium channels critical for exercise mechanotransduction (29). Calcium binding to calmodulin activates CaMKKb and AMPK, thereby initiating autophagy via FOXO3a and AMPK-ULK1 signaling pathways. AMPK inhibits mTORC1 and phosphorylates Beclin1, thereby diminishing inflammation, alleviating osteoarthritis, and preventing apoptosis (30). The AMPK-mTOR signaling pathway in the nervous system facilitates cardiac remodeling, cardiovascular function, brain adaptation, synaptic plasticity, exercise resilience, and mechanotransduction induced by exercise (31).

### 3.3 Piezo 1 and AMPK: inverse mechanical force transmission

Exercise-induced shear stress in atherosclerosis elevates Piezo1 expression, inhibits autophagy via YAP signaling activation, and facilitates nuclear translocation, underscoring the significance of physiological alterations in blood flow (32, 33). The cardiovascular system is protected and changes in brain tissue stiffness may be detected by exercise-induced Piezo1, which also improves vascular wall shear stress, blood flow optimization, Ca2 + signaling activation, and AMPK-dependent autophagy (34). In 5 FAD mice, piezo1 activation enhances neural plasticity, reduces the pathology of Alzheimer’s disease, and promotes autophagy by engulfing and breaking down amyloid-beta (35).

## 4 Bioactive compounds associated with physical activity

### 4.1 Myokines

Myokines, originating from skeletal muscle, are essential for the health benefits associated with physical exercise. They exert localized and pleiotropic effects. Inactivity diminishes myokine response, potentially associated with chronic diseases. Myokines improve health by secreting humoral factors that may facilitate the browning of adipose tissue (25). Myokines, generated during physical activity, are bioactive compounds such as IGF-1, VEGF, irisin, and lactate, which exert both local and systemic influences on organs including the heart, brain, and skeletal muscle. Exercise-induced metabolic adaptation, tissue repair, and general health depend on myokines, which can cross the blood–brain barrier and interact with particular tissue receptors (25, 36).

### 4.2 Growth factors

Exercise prompts muscle cells to generate growth factors such as IGF-1, which suppresses autophagy through the PI3K/Akt/FOXO and PI3K/Akt/mTOR paths, consequently enhancing the production of vital growth factors (37). Skeletal muscle adapts to lipids during fasting and exercise to maintain glycogen reserves and regulate blood glucose levels. AMPK is the primary sensor for these adaptations, converting information into SIRT1-mediated deacetylation of PGC-1α and FOXO transcriptional regulators. Insufficient AMPK activity undermines SIRT1-mediated responses, compromising PGC-1α deacetylation and mitochondrial gene expression. VEGF, an essential growth factor, facilitates angiogenesis and blood circulation in muscle tissue during exercise, thereby indirectly fostering the autophagic process by removing waste and dysfunctional cellular components, which are critical elements of autophagy (38).

### 4.3 Irisin

Irisin is a hormone-like myokine that is released into the bloodstream during physical activity after being cleaved from protein five, which contains the fibronectin type III domain. It modulates apoptosis, inflammation, and oxidative stress, and promotes autophagy, a process akin to hormonal function. Irisin increases the expression of LC3-II and p62 in the circulatory system, mitigating stress-induced myocardial hypertrophy (39). It facilitates autophagy in the musculoskeletal system via the Atg12-Atg5-Atg16L complex. By stimulating Wnt/β-catenin signaling, AMPK activity, and autophagy, it maintains skeletal integrity and reduces the buildup of β-amyloid proteins (40).

### 4.4 Lactate

The production of lactate, a necessary metabolite that regulates autophagy through signaling pathways including reactive oxygen species, ERK1/2, mTOR, and p70S6K, is stimulated by high-intensity exercise. Under the stress of exercise, lactate promotes Vps34 lactylation and aids in lysosomal degradation, maintaining muscle homeostasis. In diabetics, high-intensity interval training (HIIT) lowers blood glucose levels by stimulating autophagy and activating the ERK/Ribosomal protein S6 kinase, 90 kDa pathway (41). Studies indicate that polyphenols, especially quercetin, may mitigate post-exercise muscle damage, rendering them a beneficial nutritional approach for athletes. These compounds facilitate muscle recovery, improve exercise performance, and regulate inflammatory pathways, thereby enhancing mitochondrial function. They may further augment the advantages of lactate by facilitating autophagy (42).

## 5 Biological factors and various exercise parameters influencing autophagic responses

Exercise profoundly influences autophagy, a crucial modulator of the immune response, with its characteristics, intensity, and duration markedly affecting health and disease. Incorporating exercise regimens activates both aerobic and anaerobic systems; however, excessive training may result in maladaptive responses. Weight bias affects exercise identity, resulting in either adaptive or maladaptive behaviors. Individuals possessing a robust exercise identity and weight bias are more inclined to partake in maladaptive behaviors. Subsequent investigations ought to examine this correlation (43). The stages of life, sexual dimorphism, body composition, and muscle fiber types all have a significant impact on the autophagic response to physical exercise (44).

## 6 Different types of exercise in association with autophagy

**Figure 2:**
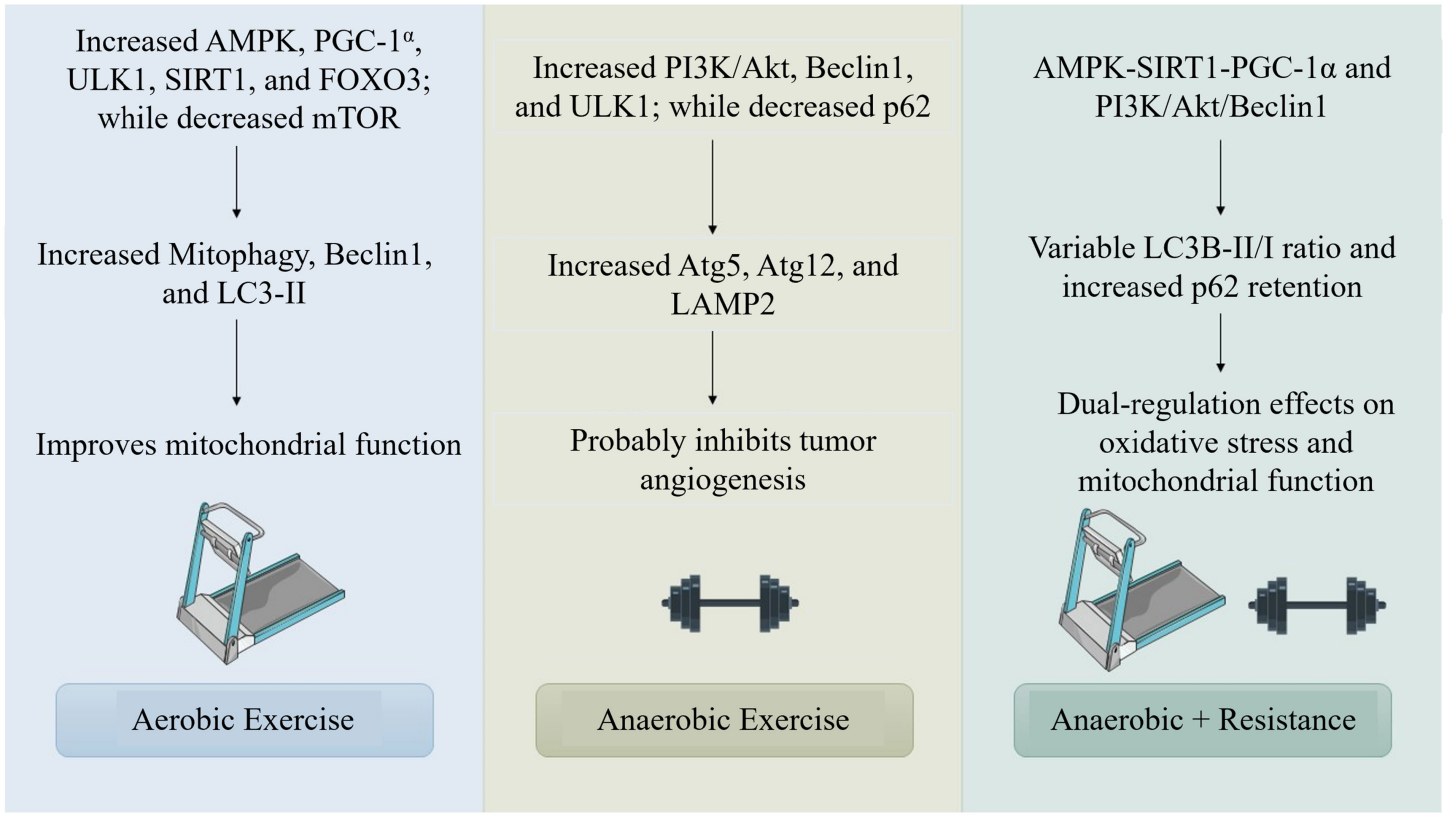
Comparison of molecular pathways activated by aerobic, anaerobic, and combined exercise modalities in regulating autophagy reveals diverse and complementary mechanistic roles. Aerobic exercise primarily stimulates the AMP-activated protein kinase (AMPK) pathway and its downstream effector peroxisome proliferator-activated receptor gamma coactivator 1-alpha (PGC-1α), which collectively enhance mitochondrial biogenesis and energy metabolism. This augments autophagic activity as a cellular adaptation to increased energetic demand. Mechanistically, AMPK activation inhibits the mechanistic target of rapamycin (mTOR), a central negative regulator of autophagy, thereby enabling the initiation of autophagic flux, particularly in skeletal muscle and cardiac tissues.

Exercise, categorized as aerobic or anaerobic, encompasses metabolic processes such as oxidative metabolism and glycolytic pathways, with exercise-adapted molecules augmenting signaling pathways such as AMPK, PI3K/Akt, mTOR, Sirt1, and CaMKs, for autophagy (41, 42) (Figure 2).

### 6.1 Combination exercise

The research indicates that the integration of aerobic and resistance training, a type of exercise, produces differing impacts on autophagy. A preclinical tumor study employed resistance training on a 1-meter ladder featuring rungs spaced 1.5 centimeters apart and inclined at an angle of 85 degrees (45). It has been shown that the training regimen, which consists of three sets of two repetitions on a treadmill for 25 min, is effective in inhibiting the growth of tumors by reducing autophagy, lowering the LC3B-II/I ratio, and maintaining high levels of p62 expression. Exercise evaluations and adrenergic stimulation do not significantly increase hepatic autophagy levels or autophagy responses, according to research on aged mice and clinical trials (46). According to the research, factors like type, intensity, and duration all influence how combined exercise affects autophagy, suggesting that longer or more intense exercise may be necessary to increase autophagic activity (47).

### 6.2 Anaerobic exercise

There is a lack of knowledge about how resistance training and other anaerobic processes affect autophagic regulation, as evidenced by the paucity of research on autophagy triggered by anaerobic exercise. Through pathways like PI3K/Akt, PINK1, Beclin1, and ULK1, resistance training increases autophagic activity (48).

### 6.3 Aerobic exercise

**Figure 3:**
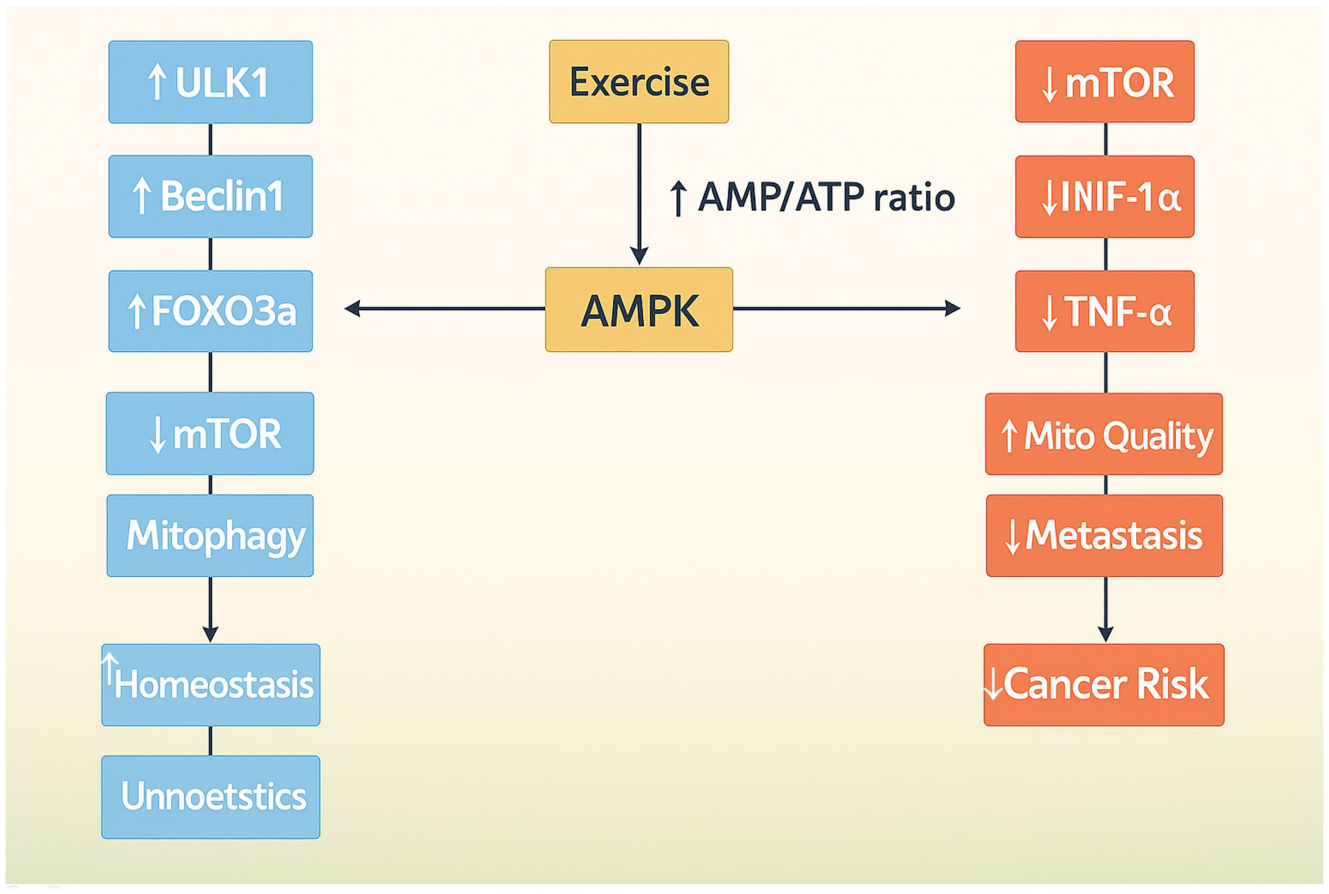
Illustration of AMPK as a central node in mediating both autophagy and anti-cancer effects during aerobic exercise. Through the inhibition of mTOR and regulation of glucose and oxygen homeostasis, AMPK contributes to metabolic reprogramming and tumor growth suppression.

Aerobic exercise significantly influences cellular homeostasis and adaptation through the activation of autophagy (49). AMPK modulates cellular energy metabolism, which is associated with the progression of cancer cells. It functions as a tumor suppressor by inhibiting mTOR and promoting cellular autophagy. AMPK can enhance autophagy by activating ULK1 and suppressing the proliferation of breast cancer cells. Nonetheless, this may result in drug resistance during subsequent stages of the tumor. AMPK is also a key protein in the TME, having a bidirectional effect on tumor growth, promoting glucose metabolism and angiogenesis. Gene knockouts can impede tumor proliferation, particularly in preneoplastic lesions. Nevertheless, AMPK activity is diminished during energy sufficiency, influencing glycolysis and lipogenesis. Aerobic exercise markedly increases AMPK activity, facilitating angiogenesis and suppressing tumor cell metastasis. Liver kinase B1 (LKB1) inhibits mTOR by activating AMPK under low ATP conditions. The reduction of ATP during exercise increases the AMP/ATP ratio and activates AMPK through LKB1. In pathological conditions, hypoxia stimulation in the tumor microenvironment activates AMPK, thereby inhibiting angiogenic factors. Aerobic exercise intervention is thus advised during the early phase of tumorigenesis (Figure 3) (50, 51).

#### 6.3.1 Running

Running is a prevalent form of exercise in clinical training and autophagy research, utilizing treadmill models in preclinical studies. This form of exercise improves the AMP/ATP ratio, thereby activating P2X7 receptors and inducing AMPK activation. AMPK activates ULK1 kinase, thereby initiating autophagy, through the inhibition of mTOR activity (52). Running influences NAD + and AMPK concentrations, activating Sirt1, which promotes autophagy via FOXO3 and BNIP3, consequently impacting downstream targets. AMPK augments PGC-1a activity, which in turn elevates PINK1 and Parkin levels in mitophagy regulators, thereby facilitating mitochondrial quality control via autophagy and improving mitochondrial function (53, 54).

#### 6.3.2 Swimming

AMPK, Akt, mTOR, FABP1, Beclin1, HSP70, and microRNAs are among the key signaling pathways that swimming affects to affect autophagic activity. AMPK activation promotes autophagy by elevating PGC-1a and FOXO3a levels, whereas swimming inhibits FABP1, thereby activating autophagy (55). The exercise-autophagy axis encompasses micro-RNAs that regulate autophagy proteins such as ULK1 and Beclin1 (56).

#### 6.3.3 Cycling

AMPK activation, mTOR inhibition, and ULK1 stimulation are some of the molecular pathways through which cycling triggers autophagy. Its importance in cellular homeostasis and stress response is highlighted by the possibility that it may also promote autophagy by activating Tumor Protein 53 (p53) (57, 58).

#### 6.3.4 Exercise programs: acute and chronic

Chronic exercise, also known as long-term exercise, includes planned, repetitive, and sustained physical activity over a longer period, while acute exercise is short-term, fleeting physical activity that does not involve regular or prolonged training (59). Acute exercise is brief, inducing immediate physiological stress, whereas chronic exercise persists for several weeks and results in temporary stress. Extended physical activity results in enduring physiological adaptations such as enhanced muscular strength and improved cardiorespiratory endurance (60, 61). The effects of autophagy differ based on exercise duration, with acute and chronic regimens yielding distinct outcomes in various tissues (Table 2). Acute exercise may either augment or diminish autophagy, while chronic exercise predominantly enhances it in particular tissues. Research indicates that chronic exercise enhances autophagy in rodent cardiac muscle, cycling promotes it in skeletal muscle, while treadmill exercise diminishes it in the liver (62). Chronic exercise consistently enhances autophagy in essential tissues such as skeletal muscle, cardiac muscle, and brain tissue, as demonstrated by increased autophagy in murine models. Autophagy is an essential regulator of central nervous system aging and neurodegeneration, maintaining neuronal health and survival by transporting organelles and toxic substances to lysosomes. Autophagic responses to exercise differ based on intensity, duration, and tissue-specific adaptability, with intense, brief exertions stimulating autophagy as a result of cellular stress. Prolonged exercise causes long-lasting changes, including increased mitochondrial biogenesis and cellular robustness, which lead to autophagic activation that continuously removes damaged organelles and maintains homeostasis. Particular tissue responses signify unique metabolic demands and molecular pathways, exemplified by the differing autophagic adaptation mechanisms of cardiac and skeletal muscles (63). Cardiomyocytes, as terminally differentiated cells, are essential for blood circulation and demand substantial energy, rendering them highly reliant on mitochondrial function for sustained contractile activities. In the heart, mitophagy is crucial because it removes damaged mitochondria, preserving regular energy metabolism and cellular respiration (64, 65). This is especially true for cardiomyocytes, which have high energy needs and mitochondrial activity. Autophagy is vital for protein metabolism and energy provision in skeletal muscle, which is essential for daily activities and movement, as it is closely linked to the health and function of cardiomyocytes (66). Exercise increases the body’s metabolic capacity, and autophagy is crucial for removing aged proteins and mitochondria, which promotes the growth of new mitochondria and muscle mass. Research has shown that chronic exercise is more important than acute exercise because it consistently increases autophagy (67).

**Table 2:** The molecular difference of autophagic biomarkers based on exercise type and duration, in various tissues.

Exercise type	Exercise duration	Tissue type	Molecular mechanism related to autophagic markers	References
Aerobic exercise	Acute Exercise	Human Skeletal Muscle	Acute exercise, training, and insulin stimulation can diminish the LC3-II/LC3-I ratio, a commonly utilized autophagy marker. The activation of AMPK during exercise is inadequate for regulating muscle autophagosome levels, whereas mTORC1 signaling through ULK1 probably governs insulin’s inhibitory effect on autophagy.	(136)
Aerobic exercise	Acute high-intensity interval training (HIIT) and moderate-intensity continuous training (MICT)	Human Skeletal Muscle	Acute HIIT and MICT induce alterations in autophagy markers, with the exercise-induced autophagy response differing across tissues and between genders.	(160)
Aerobic exercise	High-Volume HIIT	Human and rat Skeletal Muscle	Exercise-induced changes in autophagosome content markers differ between rodents and humans, and exercise-induced decreases in LC3B-II protein levels do not reflect autophagy flux levels.	(46)
Aerobic exercise	Eccentric exhaustive exercise	Rat Skeletal Muscle	Basal autophagy factors p62 and Lamp-2 increased significantly 48 h after eccentric exhaustive exercise and immediately after blunt trauma. Mitochondrial autophagy factor BNIP3 did not increase after exhaustive exercise and blunt trauma, but NIX only increased after exhaustive exercise	(161)
Aerobic exercise	High-intensity exercise	Human Skeletal Muscle	Macroautophagy and chaperone-mediated autophagy pathways are strongly activated by high-intensity exercise, regardless of PO2, and oxygenation is necessary to revert these signals to pre-exercise values. PHAF1/MYTHO emerges as a pivotal exercise-responsive autophagy marker positively associated with the LC3B-II: LC3B–I ratio	(162)
Aerobic exercise	Endurance (END), exhaustive (ET), strength (ST), and concurrent (CC) physical exercise	Rat gastrocnemius muscle, heart, and liver	The research identified alterations in mRNA expression in gastrocnemius muscle samples, with increased autophagy markers in the CC group. Heart levels diminished in the ET group, whereas liver protein levels were downregulated in the same group.	(163)
Aerobic exercise	Acute Exercise	Human Skeletal Muscle	One exercise session elevated LC3I, LC3II, and BNIP3 protein levels in human skeletal muscle, whereas 8 weeks of exercise training augmented basal levels of LC3I, BNIP3, and Parkin protein in human skeletal muscle.	(48)
Aerobic exercise	Acute Exercise	Rat Skeletal Muscle	Exercise-induced metabolic adaptations entail enhanced mitochondrial turnover, resulting in augmented degradation and biogenesis, partially regulated by PGC-1α.	(142)
Aerobic exercise	Acute Exercise	Rat Skeletal Muscle	Acute inhibition of autophagy in skeletal muscle just before exercise does not have an impact on physical performance, PRKAA1 activation, or glucose homeostasis.	(164)
Aerobic exercise	Chronic Exercise	Human Skeletal Muscle	The decline in autophagosome content suggested in human skeletal muscle during exercise is the result of a strong autophagosome degradation due to a rapid enhancement in autophagy activity, or a decrease in the activity of the system. MTORC1 signaling markedly affects insulin’s capacity to inhibit autophagy, whereas AMPK activation alone does not substantially elevate autophagosome levels during exercise.	(148)
Aerobic exercise	Chronic Exercise	Cardio myocytes	Exercise caused physiological hypertrophy influenced by Yap/Taz, autophagy, and myosin heavy chain (MHC) dynamics	(165)
Anaerobic exercise	Resistance exercise training	Human Skeletal Muscle	After a training regimen, the expression of beclin-1, Atg12, Atg16, and LAMP-2 was elevated, whereas the phosphorylation of p62/SQSTM1 and ULK-1 was diminished. Resistance training also reduced NLRP3 expression, the caspase-1/procaspase-1 ratio, Bcl-2 and Bcl-xL expression, the Bad/Bcl-2 ratio, and caspase-3 protein levels.	(166)

## 7 Insufficient autophagic response training

Muscle injury resulting from vigorous physical activity can impede daily functions. Overtraining may increase autophagy levels, which may be related to this damage and may be caused by the FOXO3/GABA type A receptor-associated protein-like 1 (GABARAPL1) signaling pathway (68). Increased skeletal muscle growth is linked to higher levels of Atrogin-1 and Muscle RING-finger protein-1 (MuRF1), two important factors that control muscle atrophy. Excessive exercise can also cause cardiac remodeling to shift from adaptive hypertrophy to harmful pathological alterations (69). Overexertion influences autophagic flux in skeletal muscle, but not in cardiac or hepatic tissue, underscoring the dual function of autophagy in overtraining and tissue-specific reactions (70).

## 8 Effects of sex metamorphosis, the composition of the body, life cycle, and muscle fiber type

### 8.1 Sexual metamorphosis

The hepatic mitochondrial adaptability and skeletal muscle performance are significantly impacted by sexual metamorphosis. This is particularly true in the liver, where autophagy is triggered by the mitochondrial biogenesis (PGC-1a) and mitophagy (BNIP3) pathways, sexual metamorphosis has a significant impact on exercise-induced autophagy, affecting skeletal muscle function and hepatic mitochondrial adaptability (71). Compared to male mice, female mice have lower autophagic flux but higher mitochondrial content and levels of autophagy-related proteins. Sex-specific variations in autophagy and adaptive responses are revealed by the requirement for daily physical activity in male mice to attain a similar mitochondrial phenotype. In skeletal muscle, the basal autophagic flux is higher in females, whereas in young male mice, exercise specifically promotes autophagy. Male skeletal muscle also exhibits increased nuclear localization of TFEB, an essential transcription factor (72).

### 8.2 Muscle fiber types

Skeletal muscle fiber types differ in their structural characteristics and metabolic capacity, which affects the autophagic response to exercise. A greater variety of fiber types, such as Type I, IIA, and IIX myosin heavy chain subtypes, are found in rodent skeletal muscle. While IIX and IIB fibers maximize anaerobic metabolism and rapid contraction, type I fibers improve endurance and energy efficiency. Intermediate fibers, or type IIA fibers, contract at moderate rates and possess oxidative and glycolytic characteristics. Different muscle fibers exhibit different patterns of autophagy; slow-twitch muscles spare slow-twitch muscles, while fast-twitch muscles, such as the gastrocnemius, are affected by starvation-induced autophagy (73). Autophagy is more strongly induced in oxidative soleus muscles by endurance training. In skeletal muscle, prolonged exercise promotes the shift to slow-twitch fiber predominance, which in turn increases autophagic capacity. According to research, autophagy is only found in oxidative soleus (SOL) muscles and not in glycolytic extensor digitorum longus (EDL) muscles, as indicated by LC3-II expression (74). Exercise may cause changes in the type of muscle fibers because it stimulates autophagy in oxidative muscle fibers. In oxidative muscles, endurance exercise promotes autophagic repair processes, which may protect them from excessive catabolism (75).

### 8.3 Life cycle

Exercise-induced autophagy exhibits considerable variation across different life stages, especially in younger individuals, facilitating swift cellular repair, metabolic adaptation, and enhanced physiological resilience, although it remains robust in older individuals. Research conducted by Zhou, Luo, and Yao showed that older adults exhibit a diminished autophagic response attributable to the age-related decline of autophagy-associated pathways. The skeletal muscle of older mice has substantially fewer autophagy-related genes, such as Beclin-1, Atg14, and LC3, than that of younger mice (76). Exercise partially reinstates autophagic activity in aged skeletal muscle, improving muscle function and bone mass. Exercise-induced autophagy facilitates osteogenic differentiation in older individuals, whereas younger animals preserve cardiac homeostasis despite heightened myocardial apoptosis and fibrosis (77). Even in older adults, exercise can reverse age-related changes by boosting basal autophagy capacity, restoring autophagic activity, and improving cardiac function (78).

### 8.4 The composition of the body

Exercise diminishes body fat, enhances glucose and lipid metabolism, and mitigates obesity by decreasing autophagy. Obesity results in compromised cellular autophagy, characterized by elevated levels of Atg5, LC3-I, and LC3-II mRNA in visceral adipose tissue, and a positive correlation with BMI. Obesity induced by a high-fat diet in mice leads to reduced autophagy activity in skeletal muscle relative to control mice. Obese mice exhibit heightened early autophagy stages in white adipose tissue, facilitating the development of autophagosomes. Obesity can induce alterations in energy metabolism within adipose tissue (79, 80). Autophagy modulates adipocyte functionality and energy equilibrium, as evidenced by knockout mice exhibiting alterations in lipid metabolism, including diminished adipose tissue, reduced adipocyte size, and weight loss. Exercise promotes autophagy in subcutaneous adipose tissue and inhibits it in perirenal adipose tissue, whereas obesity diminishes autophagy by decreasing lysosomal quantity, acidity, and fusion. Impairments in autophagic function, a hormone predominantly synthesized by white adipose tissue and governed by the obesity gene, may obstruct the body’s capacity to sustain cellular homeostasis amid metabolic stress (81, 82). Obesity frequently results in leptin resistance, marked by increased blood leptin concentrations and diminished responsiveness, especially in the hypothalamus, which is essential for energy regulation. Due to a lack of autophagy and poor energy regulation, leptin resistance causes obesity and metabolic diseases. Although autophagic function and leptin sensitivity can be improved by physical activity, there is no concrete proof that exercise-induced autophagy and increased leptin sensitivity are related. In certain tissues, obesity modifies systemic autophagy, whereas exercise promotes autophagy, maintaining cellular homeostasis and increasing energy expenditure. Further investigation is required to comprehend the influence of body fat composition on the autophagic response to exercise (13).

## 9 Autophagy induced by exercise in maintaining health and recovering from disease

In systemic diseases where autophagy is often impaired, exercise is a rehabilitative therapy that enhances the body’s ability to break down damaged cellular components. Exercise promotes autophagy, which improves cellular homeostasis and disease resistance while preventing relapse. This process aids in preserving organ health, mitigating aging, and fostering longevity. Exercise promotes autophagy, modifies pathological processes, and restores normal organ functions in the neurological and hormonal systems, heart and blood Systems, digestive, and musculoskeletal, exercise promotes healing and maintains health (56). Exercise improves disease management and health promotion by stimulating autophagy to modulate essential signaling pathways such as BMP, FOXO, NLRP3, IGF, HIPPO, ERK, and NFjB. This mechanism modulates glycolipid metabolism, diminishing cellular senescence, oxidative stress, apoptosis, and inflammation (83).

### 9.1 System of musculoskeletal

Exercise improves muscle and bone health by increasing fiber strength, promoting bone formation, and decreasing musculoskeletal injuries. Autophagy mitigates mitochondrial dysfunction, enhances antioxidant defense mechanisms, and stimulates downstream signaling pathways (84). Exercise-induced autophagy also controls important pathways for muscle growth and degeneration. The activation of the PI3K/Akt pathway results in an elevation of anti-apoptotic proteins and a reduction of pro-apoptotic factors, concurrently inhibiting the ubiquitin ligases MuRF-1 and MAFbx. This indirectly inhibits the HIPPO pathway, mitigating muscle dysfunction linked to sarcopenia. Through the inhibition of protein degradation via the Ubiquitin-Proteasome System, the modulation of IGF-mTOR signaling and the NFjB inflammatory pathways, and the upregulation of autophagy-related molecules like Beclin-1 and Atg7, exercise reduces growth inhibitor activity. By triggering the Wnt/β-catenin signaling pathway, activating autophagy-related complexes, encouraging osteogenic differentiation and bone formation, and preventing protein accumulation, exercise helps prevent aging-induced sarcopenia (85–87). This maintains chondrocyte homeostasis and prevents misfolded proteins. Exercise improves osteopenia and cartilage degradation in arthritis by boosting the NLRP3 signaling pathway, which in turn reduces pyrophosphate toxicity and degrades inflammatory vesicles. Initial exercise treatments are beneficial for musculoskeletal conditions because they reverse muscle atrophy, restore function, and improve tissue health while controlling molecular pathways that promote strength and recovery (88).

### 9.2 The neurological system

While autophagy helps maintain neuronal homeostasis by removing dysfunctional mitochondria and amyloid-beta, exercise enhances cognitive abilities and lowers neurological disorders by encouraging synaptogenesis, axonogenesis, neuroplasticity, and neuronal proliferation. Exercise-induced autophagy enhances neuronal health by preventing the accumulation of neurotoxic aggregates, optimizing mitochondrial function, diminishing oxidative stress, and maintaining neuronal integrity via essential signaling pathways. Neurodegenerative diseases such as Parkinson’s and Alzheimer’s may be mitigated by stimulating the Akt-FOXO-mTOR pathway and Beclin1-dependent autophagy, an essential regulator of cellular catabolism. Autophagy reduces neuronal damage, improves neuronal plasticity, and minimizes neurodegeneration by eliminating neurotoxic aggregates such as amyloid-beta peptides and alpha-synuclein. In Alzheimer’s disease, exercise-induced autophagy triggers the Beclin1-dependent pathway, which raises Neuregulin 1 expression and triggers the Akt-FOXO-mTOR signaling cascade (89). Physical activity enhances ERK-RSK-cAMP-response element binding protein activity enhances antioxidant defenses, mitigates neuronal degeneration, and facilitates autophagy, rendering it essential for Parkinson’s disease, Alzheimer’s disease, and cognitive rehabilitation. Exercise improves neuroplasticity, corrects deficiencies in synaptic and axonal transport, and enhances learning and cognitive abilities. It promotes neuroprotection through autophagy, regulating stress responses and enhancing angiogenesis in ischemic injuries. In reaction to injury, p62 amplifies the ERK pathway, Atg3 alleviates stress, and the PI3K-Akt–mTOR axis is stimulated, consequently reducing neuronal necrosis, strengthening defense mechanisms, and promoting tissue recovery (90, 91).

### 9.3 Heart and blood systems

Exercise stimulates autophagy to sustain energy balance and degrade damaged proteins, thereby decreasing cardiomyocyte apoptosis and reestablishing cellular homeostasis. Mitophagy induced by exercise enhances mitochondrial quality and cardioprotection, while autophagic markers such as Beclin1 and LC3-II are elevated in myocardial ischemia and hypoxic injury cases, encouraging the breakdown of organelles and metabolic waste products. By enhancing mitochondrial ATP-sensitive K + channels, lowering ischemic injury, and promoting autophagy via the AMPK-ULK1 pathway, exercise inhibits cardiomyocyte apoptosis and lowers endoplasmic reticulum stress. Exercise increases mitochondrial oxidative capacity and ATP synthesis boosts mVps34 activity, and stimulates autophagy, all of which improve myocardial function and prevent heart failure (92, 93). These enhancements reduce cardiac stress and facilitate the heart in fulfilling its energy requirements. In patients with atherosclerosis, swimming decreases inflammatory cytokines such as MMP-9, IL-6, and sICAM-1-1, enhances autophagy by activating LC3 and Beclin1, and slows the development of aortic plaque. Myocardial metabolic function and mitochondrial biogenesis are improved by exercise training, which also improves heart and blood health. It improves cardiac output, circulation, and myocardial architecture while also increasing membrane permeability. The cardiovascular system is protected by exercise-induced autophagy, which improves mitochondrial function and breaks down malfunctioning components to aid in recovery during stress and illness (94–96).

### 9.4 Hormonal system

Myocardial metabolic function and mitochondrial biogenesis are improved by exercise training, which also improves cardiovascular health. It improves cardiac output, circulation, and myocardial architecture while also increasing membrane permeability. The cardiovascular system is protected by exercise-induced autophagy, which improves mitochondrial function and breaks down malfunctioning components to aid in recovery during stress and illness. Exercise-induced autophagy is essential for metabolic regulation and lipid clearance, supporting the health of the endocrine system, particularly in metabolic diseases such as non-alcoholic fatty liver disease (97, 98). Additionally, it promotes hepatocyte autophagy, which lowers the buildup of cholesterol and triglycerides. Myelin and C1q/TNF-related protein 5 are two components that control this process. By boosting Atg7 expression, encouraging autophagy elongation, and lowering endoplasmic reticulum stress, exercise can lessen hepatic steatosis in fatty liver conditions. Additionally, it alters the Akt signaling pathway, which triggers autophagy and inhibits the release of TNF-α. This lowers MIF levels and lessens the metabolic alterations linked to obesity. Autophagy brought on by exercise decreases the buildup of fat in the liver by triggering the AMPK pathway, which regulates glucose metabolism and disorders (99–101). Physical activity in individuals with diabetes stimulates AMPK, PGC-1a, and BNIP3 expression, reduces endoplasmic reticulum stress, and promotes autophagic function, thereby alleviating renal damage in patients with chronic kidney disease (102).

### 9.5 Malignancy and aging

In aged organisms, exercise-induced autophagy improves tissue vitality and durability by preventing cell death and lipofuscin accumulation. Through AMPK-dependent autophagy, exercise extends lifespan by modifying the FOXO/Eukaryotic signaling pathway during senescence. Exercise suppresses apoptosis, increases autophagy, lowers antiapoptotic B-cell lymphoma-extra-large proteins, and delays cellular senescence (103). Additionally, it reduces the activity of cancer cells by removing oncogenic molecules, reactive oxygen species, misfolded proteins, and damaged mitochondria. Through the preservation of cellular homeostasis, inhibition of tumor growth, and enhancement of cell viability, exercise-induced autophagy promotes cancer recovery. Research indicates that it prolongs the life of colon cancer mice by decreasing the expression of Atrogin-1 and MuRF-1 (103).

## 10 Natural polyphenols combined with exercise in cancer

Natural polyphenols, plant-derived organic compounds, have been investigated for their potential health benefits, including protection against diabetes, cardiovascular diseases, oxidative stress, neurodegenerative disorders, and aging. They can suppress cancer by modifying signaling pathways, inducing apoptosis, and obstructing cell cycle processes, ultimately eradicating cancer cells. Polyphenols regulate enzymes implicated in tumor cell growth and proliferation. They additionally impede angiogenesis, avert metastasis, and engage with DNA (104). Flavonoids, phenolic acids, and tannins are essential phenolic compounds that confer health benefits by modulating inflammatory and oxidative pathways and eradicating cancer cells. Numerous athletes endorse dietary supplements to enhance physical performance during training, with research indicating the advantageous effects of specific compounds such as quercetin, resveratrol, and polyphenolic compounds derived from grape extract or beetroot juice. Exercise and antioxidant supplements may synergistically influence cancer development and progression through their antioxidant effects, thereby enhancing treatment efficacy (105).

### 10.1 Saffron

Saffron, obtained from the stigmas of the *Crocus sativus* L. plant, serves as a herbal remedy, coloring agent, and flavoring agent, and has demonstrated efficacy in addressing various health concerns (106).

The research indicates that high-intensity interval training (HIIT) and saffron aqueous extract may lower breast cancer risk by increasing Sirtuin-1 and p53 expression in tumor tissue. The study demonstrated that HIIT and SAE can reduce tumor volume and increase the expression of anti-and pro-apoptotic proteins in mice with 4T1 breast cancer (107). Nonetheless, these treatments failed to augment apoptotic induction, despite facilitating the apoptotic pathway. Its immunomodulatory properties are facilitated by multiple mechanisms, including the modulation of innate and adaptive immunity components. The pharmacological effects of saffron are chiefly attributed to crocin and crocetin, which can influence the MAPK and NF-κB signaling pathways. It regulates the expression of genes that encode pro-inflammatory cytokines, inducible enzymes, adhesion molecules, chemokines, and acute-phase proteins. These factors are essential in regulating inflammatory processes within the immune system. Consequently, saffron and its constituents may be regarded as a promising immunoregulatory agent for the treatment of immune disorders. A study conducted by Mirzaei and associates revealed that the application of saffron, honey, and rose water over 4 weeks diminished fatigue in 75 breast cancer patients. The Jollab group exhibited a marked decrease in the Visual Analogue Fatigue Scale (VAFS), Fatigue Severity Scale (FSS), and both physical and cognitive subscales of the Cancer Fatigue Scale (CFS) in comparison to the placebo group. Nonetheless, the scores on the affective subscale exhibited no significant alteration post-intervention in either group. This indicates that saffron may serve as a potential remedy for cancer-related fatigue in women diagnosed with breast cancer (108). The analyzed articles indicate that although saffron and exercise may positively influence breast cancer cells, their combination might be less effective or yield paradoxical effects. Additional *in vitro* studies may elucidate these effects and propel the investigation forward.

### 10.2 Curcumin

Curcumin, a plant comprising 120 species, is recognized for its therapeutic properties, which include antiproliferative, anti-thrombotic, antitumor, anti-inflammatory, antihepatotoxic, diuretic, hypotensive, antimicrobial, antioxidant, and antityrosinase effects (109).

**Figure 4:**
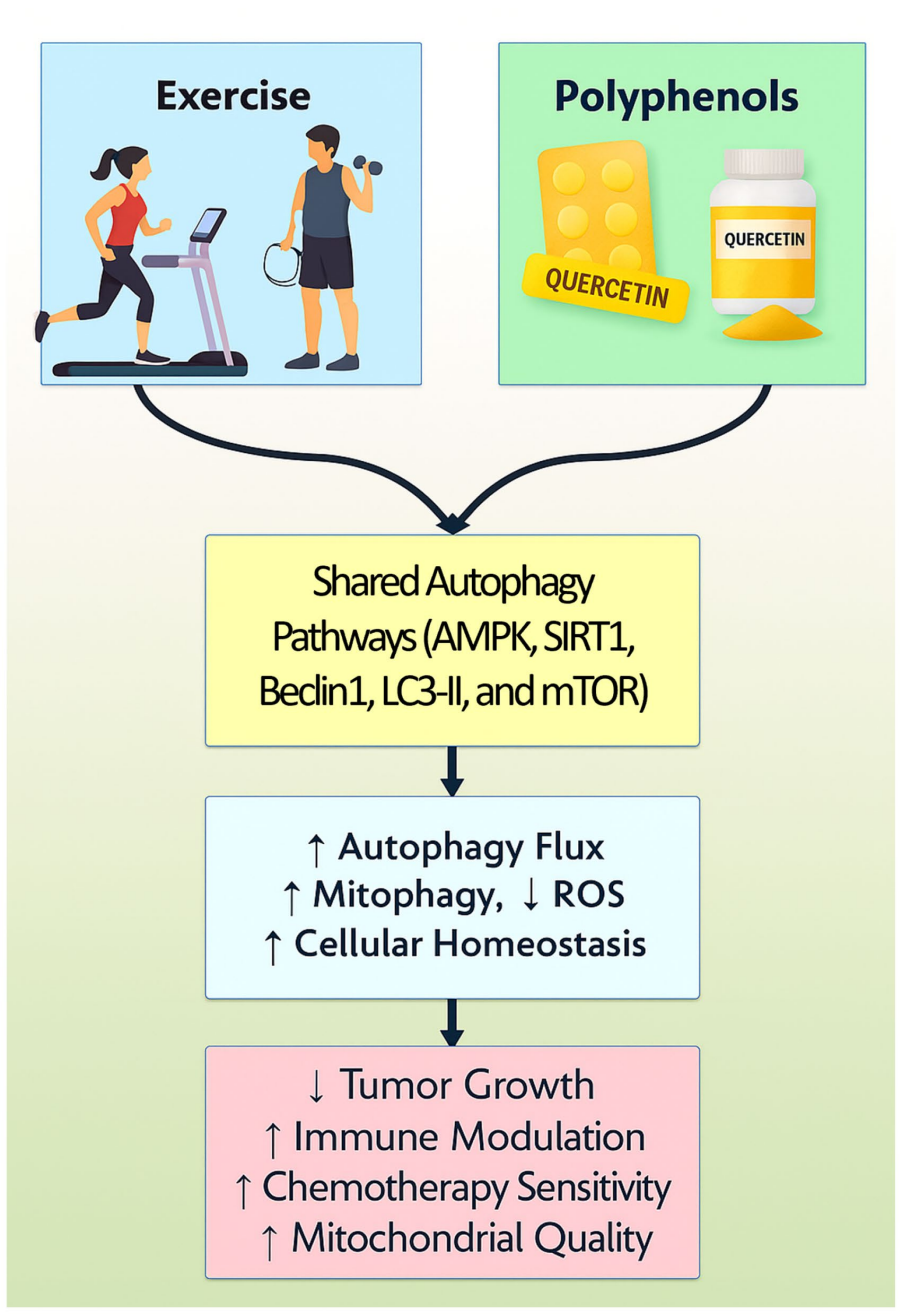
A graphical representation of the synergy between polyphenols and exercise in regulating autophagy elegantly illustrates how these two distinct but complementary stimuli converge on critical intracellular signaling networks that maintain cellular homeostasis and exert therapeutic effects. Both polyphenols and exercise activate AMP-activated protein kinase (AMPK), a master energy sensor that promotes autophagy initiation by inhibiting the mechanistic target of rapamycin (mTOR) pathway, a central negative regulator of autophagy. Concurrently, Sirtuin 1 (SIRT1), a NAD^+^-dependent deacetylase modulated by both interventions, enhances autophagic flux by deacetylating key transcription factors such as FOXO and autophagy-related proteins, thus facilitating tumor cells quality control.

Guo et al. discovered that the conjunction of curcumin treatment and swimming exercise markedly diminished breast cancer by influencing signaling pathways such as IL-17, calcium, PI3K-Akt, and Wnt (110). The combined effects of curcumin and exercise also influenced amino sugar and nucleotide sugar metabolism (111). The research indicates that endurance training combined with curcumin may improve tumor suppression. Research indicates that aerobic exercise and curcumin do not substantially mitigate oxidative stress in cancerous mice; however, they do significantly affect gene expression in mice with breast cancer (Figure 4) (111).

Aerobic exercise training diminished high-sensitivity C-reactive protein and PTX3, concurrently lowering body fat percentage and BMI, independent of curcumin supplementation. An 8-week exercise program incorporating curcumin may reduce inflammatory markers (112). Research by Moghiseh et al. demonstrates that aerobic exercise and curcumin nano micelles can mitigate the impact of doxorubicin on cardiac tissues in breast cancer patients. Aerobic exercise diminishes the expression of CAS3, CAS9, and BAX genes, whereas curcumin supplementation enhances BCL2 gene expression (113). The combination of curcumin and physical activity diminishes tumor proliferation and decreases Il4 and Stat-6 gene expression. A five-week endurance training regimen combined with curcumin is more efficacious in cancer treatment than non-pharmacological methods alone. The protective effects of curcumin against chemotherapy-induced side effects in breast cancer patients have been confirmed (114). Hemati and colleagues’ clinical trials validated curcumin’s protective properties against chemotherapy’s adverse effects in breast cancer patients. Curcumin supplementation was found to be advantageous in countering tamoxifen-induced non-alcoholic fatty liver disease, indicating its potential as a preventive adjunct to tamoxifen therapy (115).

### 10.3 Quercetin

Quercetin, a natural compound, possesses potential therapeutic applications for conditions such as diabetes, gouty arthritis, allergies, hyperuricemia, obesity, and cancer by inhibiting cancer progression, enhancing cell membrane integrity, and modulating autophagy (116). Quercetin influences multiple signaling pathways related to cell proliferation, survival, and apoptosis, including VEGF, NF-κB, and Akt/mTOR. Studies indicate that aerobic exercise and quercetin supplementation can diminish TIE-2 and VEGF-A expression in breast cancer models. This indicates that the combination may inhibit tumor angiogenesis (117). A study demonstrated that physical exercise markedly diminished tumor progression in a murine model, yielding a 75% reduction in the placebo group and a 40% reduction in the quercetin group. Additional research is required to substantiate these findings (118). Additional research is required to thoroughly understand the synergistic effects of quercetin and exercise training in cancer, encompassing optimal combinations, mechanisms, and long-term advantages. Quercetin and exercise training may substantially diminish tumor size, improve survival rates, and elevate the quality of life for cancer patients. However, the research indicates that the effects of quercetin on human subjects remain unverified, necessitating additional investigation for clinical application. The authors propose that the integration of quercetin with various training modalities may yield greater benefits or efficacy (118).

### 10.4 Daidzein

Daidzein, an isoflavone present in soy, along with exercise training, may effectively combat breast cancer by enhancing natural killer (NK) cell activity and inducing apoptosis in cancer cells. When integrated, these interventions can efficiently activate NK cells and trigger apoptosis. A study by Wang et al. revealed that consistent exercise and daidzein can markedly inhibit breast cancer proliferation in BALB/c mice. The combination also increased epinephrine and IL-6 levels, enhancing natural killer cell activity and inducing apoptosis in cancer cells (119). The study indicates that this combination could serve as an effective approach to breast cancer prevention and treatment; however, additional research is required (120).

### 10.5 Gallic acid and kaempferol

The research indicated that chemotherapy diminished JAG1 gene expression, whereas supplementation with Gallic acid and Kaempferol, along with aerobic exercise, significantly lowered its expression in both breast cancer and cancer-chemotherapy cohorts. The expression levels of BDNF and NGF genes were elevated in the cancer group receiving chemotherapy combined with supplements and chemotherapy combined with aerobic exercise, with BDNF and NGF genes exhibiting a significant increase relative to other groups. They concluded aerobic exercises and supplements declined the side effects of paclitaxel and improved the neurogenesis (121). Endurance training diminishes tumor growth and development by lowering the expression of genes such as HIF1α and VEGFα, whereas gallic acid and kaempferol regulate these genes and affect other cancer-associated genes (121). Owing to uncertainties, the routine clinical application of Gallic acid and Kaempferol is not advised, and their prospective role in clinical contexts remains conjectural (122).

### 10.6 Green tea

Catechins can mitigate muscle damage and oxidative stress in senescence-accelerated mice. Green tea extract augments physical performance in both animals and humans by enhancing endurance and lipid metabolism. Research indicates that EGCG can stimulate fat oxidation genes in the muscle mitochondria of mice subjected to a high-fat diet (123). Concentrated green tea supplements rich in catechins and caffeine can elevate daily energy expenditure in humans. These findings indicate that green tea extract may alleviate the impact of exercise on muscle health. Green tea extract has been shown to enhance fat oxidation and insulin sensitivity during moderate exercise (124). Nonetheless, short-term EGCG supplementation can result in elevated levels in adults. A controlled experiment demonstrated no notable effect on lipid and energy metabolism, inflammatory markers, or oxidative stress indicators. A study revealed no significant alterations in biomarkers following 640 mg of green tea catechins, indicating an inadequate dosage for alleviating oxidative stress and muscle damage; however, augmented aerobic exercise and green tea extract may impede prostate cancer (125). The research included cancer-afflicted rats and a healthy control cohort. A study indicates that green tea extract markedly diminishes prostate cancer risk in rats by reestablishing the equilibrium between pro-oxidants and antioxidants, involving rats subjected to HEGT treatment alone or in conjunction with aerobic exercise (126). Research indicated that aerobic exercise and green tea extract can diminish levels of cyclooxygenase-2 (COX-2), NF-kB, and P53 in the prostates of rats. The rats were categorized into six groups: healthy, cancer, low to moderate-intensity exercise, green tea extract, cancer training with green tea extract, and sham (127). Post-mortem analysis of prostate tissues revealed elevated NF-kB levels in the CCt group and diminished p53 levels in the CTr, CEx, and CTr + CEx groups, indicating that a regular intake of green tea may contribute to the reduction of these levels (128).

Physical activity is associated with cancer, as angiogenesis influences blood vessel formation. The combination of exercise training and plant-derived phytochemicals may aid in cancer prevention. Moderate aerobic exercise is more efficacious in suppressing angiogenesis markers in tumor tissue. MMPs, including MMP-9 and MMP-2, play a role in cancer cell invasion, tumor growth, and the facilitation of metastasis. Endothelial cells can selectively express and activate matrix metalloproteinases (MMPs), initiating angiogenesis and the angiogenic switch, highlighting the potential of combining exercise with plant-derived phytochemicals in cancer treatment (129). The study by Khosravi et al. investigated the effects of aerobic exercise and green tea extract on MMP-2/−9 and VEGF levels in both healthy rats and prostate cancer patients. The results indicated no significant differences in MMP-2, MMP-9, or VEGF levels between the healthy and cancer groups. The study indicates that additional research should concentrate on the regulation of tumor dissemination and angiogenesis concerning physical activity and antioxidant use (130).

### 10.7 Resveratrol

Resveratrol, a naturally occurring polyphenolic compound, is an antitoxin produced by plants in reaction to external stimuli. It is found in grapes, mulberries, cranberries, and peanuts, and has received significant attention for its cancer-preventive and anti-cancer properties in recent years. Research demonstrates that resveratrol can induce autophagic cell death through the Ca2+/AMPK-mTOR signaling pathway, leading to the death of human non-small cell lung cancer cells (A549). It can also induce apoptosis in human ovarian cancer (OVCAR-3) cells, an effect that is reduced by the autophagy inhibitor chloroquine. Resveratrol can induce autophagy in SKOV3 human ovarian cancer cells and inhibit apoptosis. However, in conjunction with the autophagy inhibitor 3-methyladenine (3-MA), resveratrol markedly increases cell apoptosis by inhibiting autophagy, suggesting that autophagy induced by resveratrol may protect SKOV3 cells. Resveratrol can stimulate autophagy and apoptosis in cisplatin-resistant human oral cancer CAR cells, whereas 3-MA obstructs autophagosome fusion and enhances CAR cell viability (131–133). Resveratrol supplementation modulates inflammation, metabolism, glucose and lipid metabolism, and muscle atrophy by enhancing AMPK activity, decreasing protein degradation, and inhibiting NF-κB; however, its effects *in vivo* remain contentious. Recently, the research demonstrated that resistance training and resveratrol supplementation significantly diminish tumor volume via mTORC1 and AMPK signaling pathways, leading to decreased phosphorylation and activation of factors and carcinogenic markers (134). Additional research is required to enhance confidence. A study demonstrated that RSV synergistically amplified the anticancer effects of DTX in prostate carcinoma LNCaP cells, resulting in heightened apoptosis and necroptosis. This indicates RSV as a potential adjuvant for DTX therapy in prostate carcinoma.it has been also determined that resveratrol, diminished A549 cell viability in a concentration-dependent manner and exhibited a synergistic effect with cisplatin and carboplatin, potentially facilitating apoptosis via autophagy and elevating reactive oxygen species levels (133).

## 11 Conclusion and future perspectives

The interplay between polyphenols and exercise in modulating autophagy presents a promising, non-invasive approach to improving health outcomes in both cancer patients and healthy individuals. By targeting key molecular pathways such as AMPK/mTOR, PI3K/Akt, and SIRT1/FOXO, polyphenols and exercise work synergistically to regulate cellular homeostasis, reduce oxidative stress, and modulate inflammatory responses. This review highlights the emerging evidence that polyphenol supplementation, combined with regular physical activity, can enhance autophagic flux, ultimately contributing to cancer prevention, improved treatment responses, and overall metabolic health. However, despite promising preclinical and clinical studies, several knowledge gaps remain. Future research should focus on a few approaches. Determining the optimal doses and bioavailability-enhancing strategies for polyphenols to maximize their autophagy-inducing effects in cancer patients and healthy populations. Also, the role of individual genetic and epigenetic variations in autophagic responses to polyphenols and exercise should be investigated to develop personalized therapeutic strategies. In addition, well-designed, large-scale clinical trials are needed to validate the efficacy of combined polyphenol-exercise interventions in cancer prevention and treatment. Further elucidating the precise molecular mechanisms by which polyphenols and exercise modulate autophagy across different cancer types and physiological states. Furthermore, exploring the long-term health benefits and potential risks of sustained polyphenol intake alongside exercise, particularly in aging populations and cancer survivors. In conclusion, integrating polyphenols with exercise holds significant potential for enhancing autophagy and promoting cellular resilience. With further research and clinical validation, this dual approach may offer an effective, accessible, and non-toxic strategy for cancer management and health optimization.
